# Assessing biomarkers of exposure to carcinogens associated with combustible cigarettes, electronic cigarettes, and heated tobacco products: a systematic review and meta-analysis

**DOI:** 10.3389/fphar.2025.1630961

**Published:** 2025-07-29

**Authors:** Yue Cao, Lin Zhang, Mengqi Yang, Jiaxuan Li, Xi Chen, Fangzhen Zheng, Jianqiang Zhang, Xiao Xu, Xiaona Liu

**Affiliations:** ^1^ Department of Health Sciences, Smoore Tech Research Institute, Shenzhen, Guangdong, China; ^2^ Department of Epidemiology and Preventive Medicine, The School of Public Health and Preventive Medicine, Monash University, Melbourne, VIC, Australia; ^3^ Suzhou Industrial Park Monash Research Institute of Science and Technology, Monash University, Suzhou, China; ^4^ Department of Pharmaceutical Science, Monash University, Melbourne, VIC, Australia; ^5^ Department of Life Science, Imperial College London, London, United Kingdom; ^6^ Department of Behavioral Sciences, Centre for Substance Use Research, Scotland, United Kingdom

**Keywords:** Biomarkers, exposure assessment, carcinogens, combustible cigarettes, electronic cigarettes, heated tobacco products, meta-analysis

## Abstract

**Introduction:**

There is growing global concern about the emissions of harmful and potentially harmful constituents (HPHCs) associated with electronic cigarette (EC) and heated tobacco product (HTP) use. This systematic review evaluates biomarkers of exposure (BoEs) for carcinogens in individuals who switched from combustible cigarettes (CCs) to either ECs or HTPs.

**Methods:**

A literature search was performed across PubMed, Ovid, and Web of Science for randomized controlled trials (RCTs) published from January 2013 to March 2024. Data synthesis was conducted using a random-effects meta-analysis, calculating ratios of means (RoMs) to compare biomarker concentrations among individuals who completely switched to ECs or HTPs, those who abstained from smoking, and those who continued smoking CCs.

**Results:**

Our analysis included 18 RCTs, examining 14 BoEs for FDA-identified carcinogenic HPHCs. Compared to continual CC smokers, individuals who completely switched to ECs demonstrated significantly lower exposure to eight carcinogens (i.e., 2-AN, 4-ABP, HEMA, MHBMA, NNAL, NNN, S-PMA, o-Tol; RoMs range: 0.031–0.461). Those who fully transitioned to HTPs showed significantly lower exposure to 12 carcinogens (i.e., 1-AN, 2-AN, 3-HMPMA, 3-OH-B [a]P, 4-ABP, CEMA, HEMA, MHBMA, NNAL, NNN, S-PMA, o-Tol; RoMs range: 0.054–0.527). No statistically significant differences in carcinogenic exposures were observed when comparing smoking cessation, or full switching to ECs or HTPs for all evaluated BoEs to continuous CC smoking, except for 3-HMPMA, CEMA, and NNN.

**Conclusion:**

Individuals who completely switched from CCs to ECs or HTPs had significantly lower exposure to numerous carcinogenic HPHCs, with the degree of reduction for some toxicants potentially approaching those of smoking abstainers. However, these findings require careful interpretation, as the evidence is predominantly derived from short-term trials (5–7 days). Further research should explore the long-term health impacts of residual nicotine and tobacco-specific toxicant exposures among these switchers.

## 1 Introduction

Smoking continues to be a leading cause of preventable morbidity and mortality worldwide, significantly contributing to the public health burden. It is a major risk factor for a range of chronic diseases, including chronic obstructive pulmonary disease (COPD), hypertension, cardiovascular disease, atherosclerosis, diabetes, and various cancers. For example, around 90% of lung cancer deaths are attributable to smoking combustible cigarettes (CCs) or exposure to second-hand smoke (How Tobacco Smoke Causes Disease, 2010). Despite ongoing efforts to reduce smoking rates, particularly in high-income regions, the global prevalence remains alarmingly high, with over 1 billion smokers recorded in 2020: 32.6% of men and 6.5% of women (Dai et al., 2022). Since 1990, traditional smoking rates have significantly declined, by 27.2% among men and 37.9% among women. However, this trend is less pronounced in low- and middle-income countries, particularly in regions such as Asia and the Pacific Islands (De Silva et al., 2024). The World Health Organization (WHO) emphasizes the urgency of addressing combustible tobacco consumption, which accounts for one in ten deaths globally, resulting in approximately 8 million fatalities each year (Jafari et al., 2021). Given the severe health risks associated with tobacco use, smoking cessation is widely recognized as the most effective strategy for mitigating these dangers (Okobi et al., 2023). Nevertheless, the journey toward cessation can be challenging for many smokers. While combining pharmacotherapy with behavioral interventions has proven successful (Okobi et al., 2023), a substantial number of smokers remain unwilling or unable to stop entirely (Klemperer et al., 2023).

In 2016, the National Center for Chronic Disease Prevention and Health Promotion (NCCDPHP) Office on Smoking and Health highlighted the significant emergence of alternative nicotine delivery systems (ANDS), including electronic cigarettes (ECs) and heated tobacco products (HTPs), signaling a shift in global nicotine consumption (National Center for Chronic Disease Prevention and Health Promotion U.S. Office on Smoking and Health, 2016). ECs, also known as vapes, entered the U.S. market in 2007 as battery-operated devices that heat liquids with or without nicotine to produce inhalable aerosols. Shortly after, refined HTPs were introduced, heating processed tobacco leaves below combustion temperatures to generate potentially less harmful, yet still risky, nicotine-containing emissions compared to CCs (Farsalinos et al., 2018; Ratajczak et al., 2020). The global market for ECs surged from USD 7.8 billion in 2015 to USD 22.3 billion in 2022 (World Health Organization, 2023). Recent data shows a slight increase in EC use among U.S. adults, rising from 4.5% in 2021 to 6% in 2022, with the highest prevalence among 23- to 24- year-olds, with 22.8% currently using ECs. In the United Kingdom, EC use steadily grew from 9.1% in 2023 to 11% in 2024, reaching 5.6 million adults (Action on Smoking and Health ASH, 2024). A 2021 cross-sectional study across 14 countries found that the prevalence of current EC use ranged from 0.02% in India to 3.5% in Russia, with approximately 18.3 million adults using ECs in total (Pan et al., 2022). HTPs have also been growing in popularity globally since 2015 (Ratajczak et al., 2020). A systematic review and meta-analysis reported lifetime, current, and daily HTP use rates of 4.87%, 1.53% and 0.79%, respectively, from 2015 to 2022, synthesizing data from 42 countries/areas across the European Region (EUR), Western Pacific Region (WPR), Americas Region (AMR) and African Region (AFR) (Sun et al., 2023). Notably, lifetime HTP use significantly increased from 0.52% in 2015 to 3.91% in 2019 for the WPR and from 1.13% in 2016 to 6.98% in 2020 for the EUR. Current HTP use rose from 0.12% in 2015 to 10.57% in 2020 for the WPR and from 0% in 2016 to 1.15% in 2020 for the EUR. The rise in ECs and HTPs reflects approximately 15–20 years of innovation in nicotine delivery, driven by public health initiatives and changing consumer preferences toward perceived safer alternatives.

For individuals seeking to reduce harm or struggling to quit smoking, emerging alternatives such as ECs and HTPs present a potential pathway to manage nicotine addiction while possibly mitigating health risks. However, the complexities of long-term health outcomes cannot be overlooked. Clinical endpoints related to diseases such as cancer generally take decades to develop, and with ECs and HTPs being relatively new to the market, there is a notable absence of epidemiological data to evaluate the chronic disease risks associated with these products in the short term. Research by Rodrigo et al. suggests that ECs and HTPs may lower lifetime cancer risk significantly, yet the evidence regarding their long-term effects remains sparse, with the findings primarily addressing short-term use and indicating fewer side effects (Rodrigo et al., 2021). Similarly, Travis and colleagues emphasized the critical gap in understanding the long-term pulmonary and carcinogenic impacts of these alternatives, leaving users exposed to the corresponding risks (Travis et al., 2022). Moreover, Polosa et al. (2019) and Cho (2023). stress the need for further investigation to clarify the overall health implications of using ECs and HTPs. Without comprehensive research, the promise of these products as safer alternatives may be overshadowed by the uncertainty around their long-term consequences.

While confirming the long-term health impacts of tobacco use is challenging, assessing toxicant exposure and estimating health risks linked to alternative tobacco products to CCs over a shorter timeframe can represent a valuable approach. This can be effectively achieved through the measurement of biomarkers of exposure (BoEs). Derived from human biospecimens such as urine, blood, and breath, BoEs reveal the actual exposure to harmful and potentially harmful constituents (HPHCs) released from the smoke or aerosols of tobacco products. They also represent the internal biological interactions between the human body and these toxicants. The Institute of Medicine of the U.S. Academy of Sciences defines a BoE as “a tobacco constituent or metabolite that is measured in a biological fluid or tissue that has the potential to interact with a biological macromolecule; sometimes considered a measure of internal dose” (Hall, 2024). Consequently, BoEs provide a more nuanced assessment of health risk compared to basic exposure metrics, such as the number of CCs smoked per day. By integrating a variety of tobacco- and disease-related variables, BoEs facilitate a comprehensive comparison of the relative harm associated with different tobacco products (Hall, 2024).

The risk continuum of tobacco and nicotine products places CCs at the high-risk end, while nicotine replacement therapies (NRTs) are at the low-risk end, with the ultimate goal being smoking cessation; both ECs and HTPs occupy intermediate points along this continuum (McAdam et al., 2018). Existing evidence suggests that ECs may be less harmful than HTPs; for instance, ECs may create lower levels of toxic and cancer-causing elements compared to conventional CC smoking (Bracken-Clarke et al., 2021). Conversely, there is currently no evidence definitively illustrating that HTPs are less harmful than traditional CCs (Copyright and License information, 2020). Research indicates that a large amount of harmful chemicals present in HTPs may exceed that found in CC smoke and that HTPs can produce toxic substances that are not typically associated with traditional CCs (Thomas et al., 2024). To fully understand the implications of these non-combustible alternatives, it is crucial to measure both the relative risk of these products in comparison to CCs, as well as their absolute risks, which can potentially be assessed by comparing them to the outcomes of smoking cessation. This bidirectional assessment will provide a clearer picture of the potential health impacts of ECs and HTPs and inform public health strategies.

There exists a limited number of short-term clinical studies assessing BoEs among individuals using various forms of novel tobacco products, including ECs and HTPs (Scherer et al., 2022a; Scherer et al., 2022b). Furthermore, systematic literature reviews summarizing findings from BoE studies have predominantly focused on either ECs or HTPs separately (Banks et al., 2023; Simonavicius et al., 2019; Drovandi et al., 2020), or have combined them without direct comparisons (Akiyama and Sherwood, 2021). To address this gap and to examine the suggestions that ECs and HTPs may serve as less harmful alternatives to CCs, we aimed to summarize the differences in FDA-identified BoEs for carcinogens among individuals who had completely switched to either ECs or HTPs and those who abstained from smoking, compared to those who were continuing to smoke CCs exclusively.

## 2 Methods

Our systematic review and meta-analysis were conducted according to the Preferred Reporting Items for Systematic Reviews and Meta-Analyses (PRISMA) guidelines and registered in PROSPERO (registration number: CRD42024584572).

### 2.1 Search strategy and study eligibility

A systematic review was conducted to identify peer-reviewed randomized controlled trials published in English between January 1, 2013 and March 1, 2024. Three main databases were searched: PubMed, Ovid, and Web of Science. The general electronic search strategy was as follows: [(“e-cigarette” OR “electronic cigarette” OR “e-vapor” OR “electronic nicotine delivery system” OR “ENDS”) AND (“biomarker” OR “BOE”) AND (“randomized controlled trial” OR “RCT”)] OR [(“heated tobacco” OR “heat-not-burn” OR “HNB” OR “tobacco heating” OR “IQOS” OR “Ploom” OR “glo” OR “novel tobacco”) AND (“biomarker” OR “BOE”) AND (“randomized controlled trial” OR “RCT”)]. This search strategy was adapted to align with the Boolean operators specific to each database. Citation and reference lists were scanned to identify additional eligible studies.

Studies eligible for inclusion were RCTs that compared BoE levels between individuals who had completely transitioned from CC smoking to EC or HTP use and those who were continuing to smoke CCs exclusively. Studies investigating BoEs in mice, rats, or *in vitro* human tissue samples were excluded, as we aimed to collect BoE data directly related to human switching behaviors. Non-RCT studies, non-peer-reviewed articles, expert opinions, conference abstracts, and studies published before 2013 were also excluded, along with studies not published in English. Two authors (Y.C. and J.L.) independently conducted the database search and cross-checked each other’s list of potentially eligible studies to ensure the inclusion of all RCTs within the specified timeframe, followed by a rigorous eligibility assessment. Disagreements were resolved via discussion or consultation with a third author (X.L.) to reach a consensus.

### 2.2 Data extraction

Selected articles were categorized into three groups: (How Tobacco Smoke Causes Disease, 2010): studies comparing biomarkers between complete EC switchers and continuous CC smokers (Dai et al., 2022); studies comparing biomarkers between complete HTP switchers and continuous CC smokers; and (De Silva et al., 2024) studies comparing biomarkers between abstainers (stopped smoking CCs without any assistance, including approved NRTs) from smoking and continuous CC smokers. Data extraction was carried out by two authors (Y.C. and J.L.) using a predetermined extraction form. Data items extracted included the title, author names, author affiliations, the year of publication, the country of participant recruitment, participant demographics (i.e., age, gender ratio, and smoking habits), intervention and control groups, devices of interest and associated features, sample sizes for each group, the intervention exposure duration, and biomarkers measured. Where BoE data were available at multiple time points, we used data from the longest follow-up after assignment to the intervention or control groups.

### 2.3 Risk of bias assessment

The risk of bias for each included study was independently evaluated by two reviewers (Y.C. and J.L.) using Version 2 of the Cochrane risk-of-bias tool for randomized trials (RoB 2). This tool assesses bias in the following five domains: (a) the randomization process; (b) deviations from intended interventions; (c) missing data; (d) the outcome measurement error; and (e) the selective reporting of results (Barili et al., 2023). For each domain, the assessment results were categorized as “low”, “moderate”, or “high”. Studies were classified as having low bias if they had fewer than one domain rated as “moderate”, with all other domains rated as “low”. Studies were considered as having moderate risk if they scored “moderate” in two or more domains without any “high” concerns. Studies were deemed as having high risk if at least one domain was recorded as having a “high” risk. Any disagreements were resolved through discussion between the two scorers or by consulting a third reviewer (X.L.) to ensure consistent bias assessment. The RoB 2 assessment was visualized utilizing traffic light and summary plots generated by the Robvis tool, providing a meta overview of the overall and domain-specific risk levels for each study (Page et al., 2023).

### 2.4 Statistical analysis

BoEs that were regularly reported in the included studies and have been identified by the U.S. FDA as carcinogenic markers were selected as outcomes of interest. In several studies, the BoE values at follow-up were adjusted for baseline values and other covariates using analysis of covariance (ANCOVA) or linear mixed-effects models (LMEM). When both raw and adjusted values at follow-up were provided, adjusted values were prioritized; otherwise, raw values were used. Geometric means and their standard deviations were converted to arithmetic means for consistency in data synthesis (Higgins et al., 2008). In cases where multiple intervention or control groups existed within a single study, they were combined to create a single pairwise comparison, following established meta-analytic practices (Jain et al., 2012). Missing standard deviations (SDs) were handled using a hierarchical approach. First, SDs were calculated from 95% confidence intervals (CIs) where available, assuming a normal distribution for sample sizes *n* > 60 and a t distribution otherwise (Cumpston et al., 2019). If no measure of variability was reported, we conservatively imputed the SD using the maximum values for that specific biomarker across the same comparative groups and measurement units.

The BoE levels at baseline were synthesized using a fixed-effects meta-analysis to detect imbalance (Wewege et al., 2022), while the follow-up values were synthesized using a random-effects meta-analysis to measure the differences between intervention and control groups. Ratios of means (RoMs) with 95% CIs were calculated to compare BoE levels between individuals who had completely switched to ECs or HTPs, individuals who abstained, and those who continued smoking CCs. The heterogeneity among pooled studies was assessed using the Q-statistic and *I* ^2^ statistic. Publication bias was evaluated using funnel plots and confirmed by Egger’s regression tests for further verification. Sensitivity Analysis was conducted to assess the robustness of our findings to the SD imputation strategy by excluding all studies that required SD imputation. All the statistical analyses were performed using R software (Version 4.3.0), with statistical significance set at a p-value of <0.05.

## 3 Results

### 3.1 Search results

**FIGURE 1:**
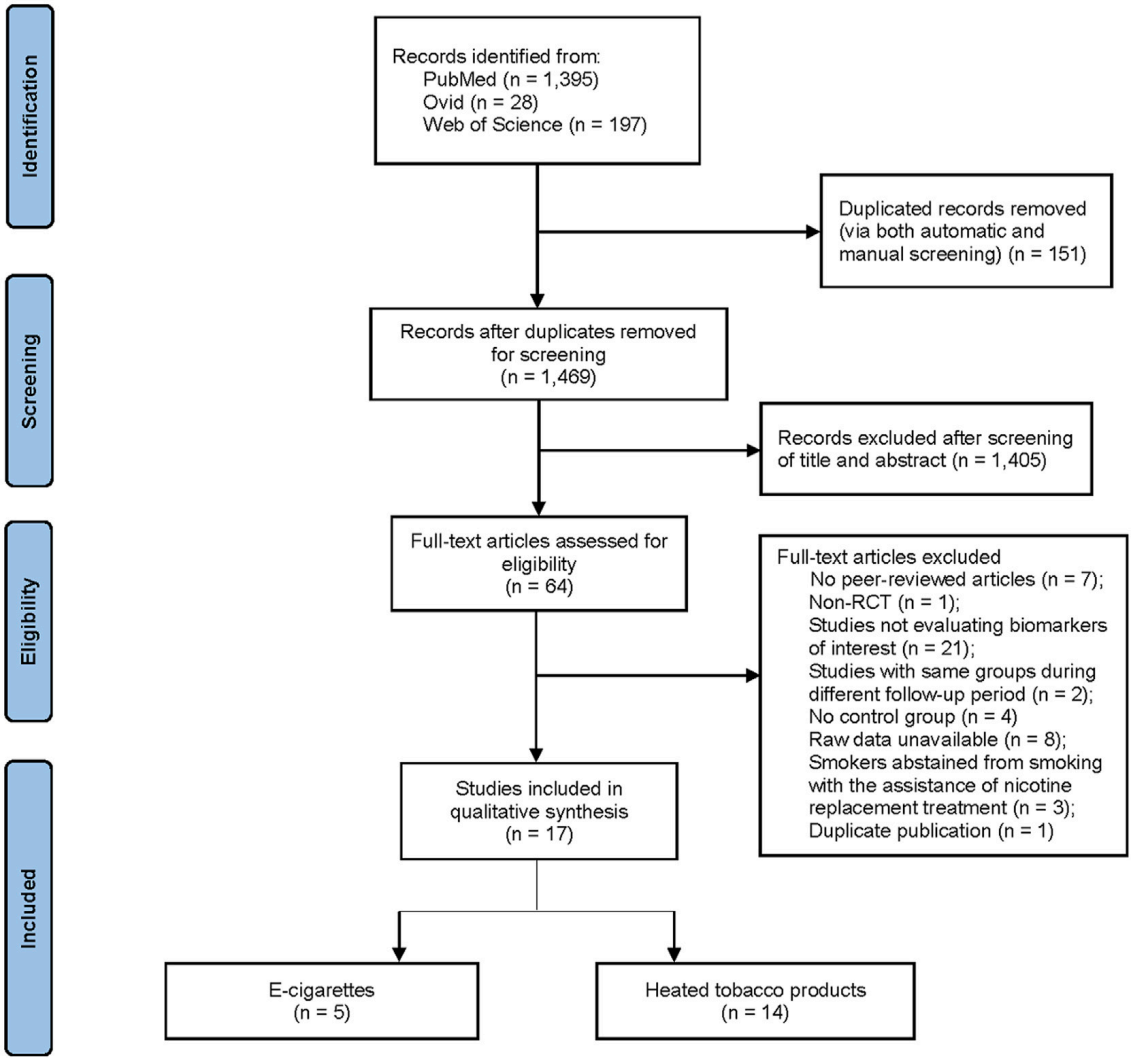
PRISMA flow chart of the selection of studies.

The initial literature search yielded 1,620 citations. After the removal of 151 duplicates through automated and manual screening, 1,469 unique studies remained. Title and abstract screening excluded 1,405 irrelevant studies in accordance with the pre-defined inclusion and exclusion criteria. Cross-reference checking did not reveal any additional articles missed by the search strategy. A subsequent full-text review was carried out to eliminate an additional 47 articles, leaving 17 eligible articles (involving 18 RCT, n = 1,648 participants) for inclusion in this systematic review and meta-analysis. Across the 17 articles, with some overlap, 14 articles evaluated HTPs (Sakaguchi et al., 2014; Haziza et al., 2016; Ludicke et al., 2016; Lüdicke et al., 2017; Lüdicke et al., 2018; Gale et al., 2019; Lüdicke et al., 2019; Haziza et al., 2020; McEwan et al., 2021; Gale et al., 2022; Morris et al., 2022; Yuki et al., 2022; Nishihara et al., 2024; Ogden et al., 2015), 5 articles assessed ECs (McEwan et al., 2021; Gale et al., 2022; Morris et al., 2022; Hatsukami et al., 2020; Edmiston et al., 2022), and 8 articles involved an abstinence group (Ludicke et al., 2016; Lüdicke et al., 2018; Gale et al., 2019; Haziza et al., 2020; McEwan et al., 2021; Yuki et al., 2022; Nishihara et al., 2024; Yuki et al., 2018). The systematic search method is shown in Figure 1.

### 3.2 Risk-of-bias assessment

The risk of methodological bias, as assessed using the ROB-2 tool, varied across RCTs (Supplementary Figure S1). The overall risk of bias was considered to be low for eight RCTs (Sakaguchi et al., 2014; Ludicke et al., 2016; Lüdicke et al., 2017; Lüdicke et al., 2018; Gale et al., 2019; McEwan et al., 2021; Yuki et al., 2022; Yuki et al., 2018), to be of some concerns for six RCTs (from five articles) (Haziza et al., 2016; Lüdicke et al., 2019; Gale et al., 2022; Morris et al., 2022; Nishihara et al., 2024), and to be high for four RCTs (Lüdicke et al., 2019; Ogden et al., 2015; Hatsukami et al., 2020; Edmiston et al., 2022). The most prevalent risks were bias due to missing data (*n* = 3), bias due to deviations from the intended intervention (*n* = 2), and outcome measurement bias (*n* = 1).

### 3.3 Biomarkers of exposure to carcinogens

Supplementary Table S1 summarizes the 14 biomarkers of carcinogenic exposure that were reported most commonly and included in the final analysis, with their corresponding biospecimens, associated HPHCs, and potentially related disease risks. All biomarkers were measured in urine, falling into four distinct chemical classes: tobacco-specific nitrosamines (TSNAs), semi-volatile organic compounds (SVOCs), aromatic amines (AAs), and polycyclic aromatic hydrocarbons (PAHs). In particular, the reported biomarkers are as follows: two TSNAs (i.e., total NNAL and NNN); seven SVOCs (i.e., CEMA, 3-HMPMA, MHBMA, SPMA, AAMA, GAMA, and HEMA); four AAs (i.e., 1-AN, 2-AN, 4-ABP, and o-Tol); and one PAH (i.e., 3-OH-B [a]P). Nine of these biomarkers (i.e., total NNAL, NNN, MHBMA, SPMA, 2-AN, 4-ABP, o-Tol, 3-OH-B [a]P, and HEMA) are classified as Group 1 carcinogens by the International Agency for Research on Cancer (IARC). Two biomarkers (i.e., AAMA and GAMA) are categorized as Group 2A, two (i.e., CEMA and 3-HMPMA) are placed in Group 2B, and one (i.e., 1-AN) is in Group 3.

### 3.4 Meta-analysis of biomarker concentrations

#### 3.4.1 TSNAs

**FIGURE 2:**
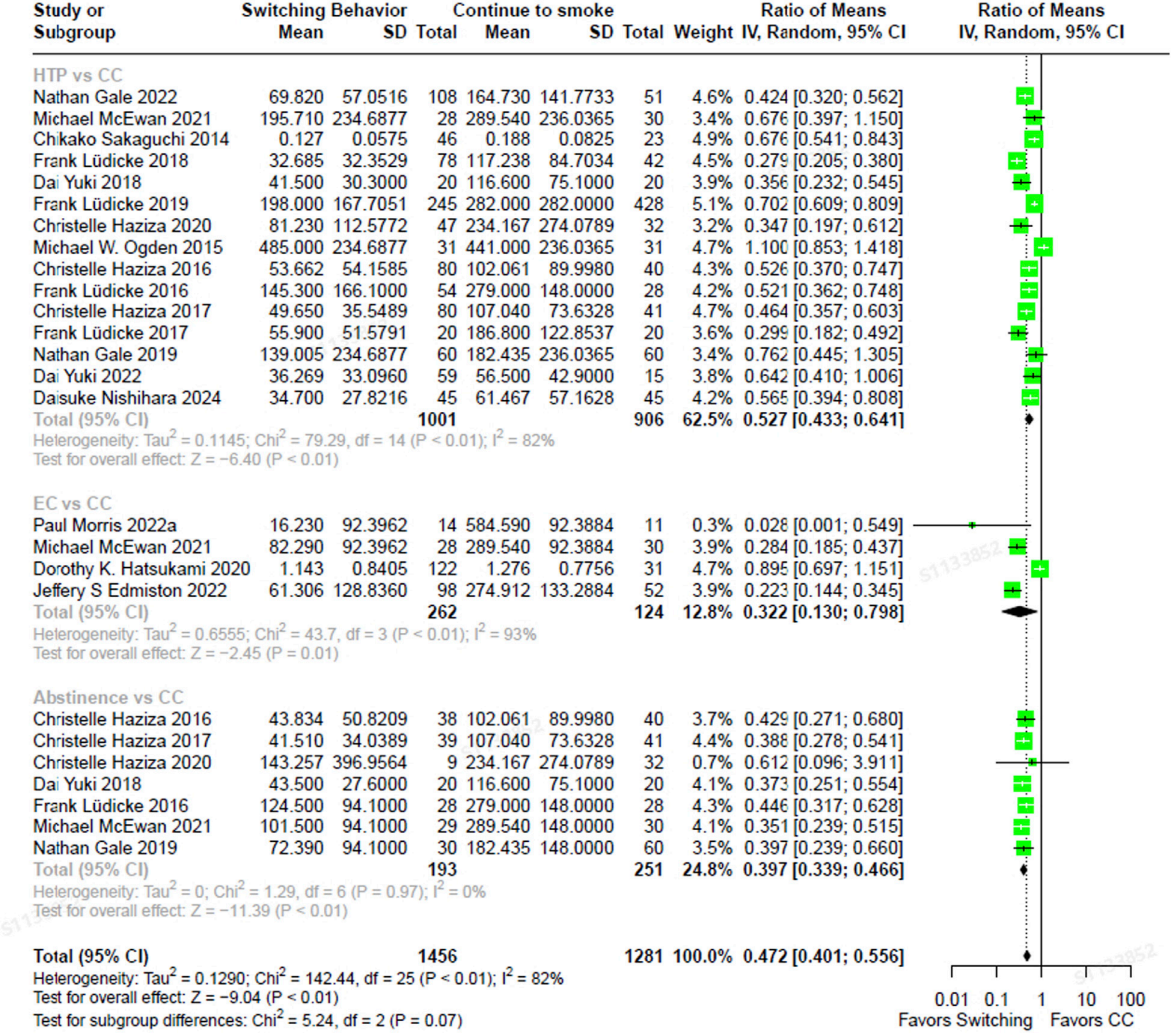
The forest plot illustrating the ratio of means in NNAL between heated tobacco product switchers, electronic cigarette switchers, smoking abstainers, versus continued combustible cigarette smokers. NNAL = total 4-(methylnitrosamino)-1-(3-pyridyl)-1-butanol.

**FIGURE 3:**
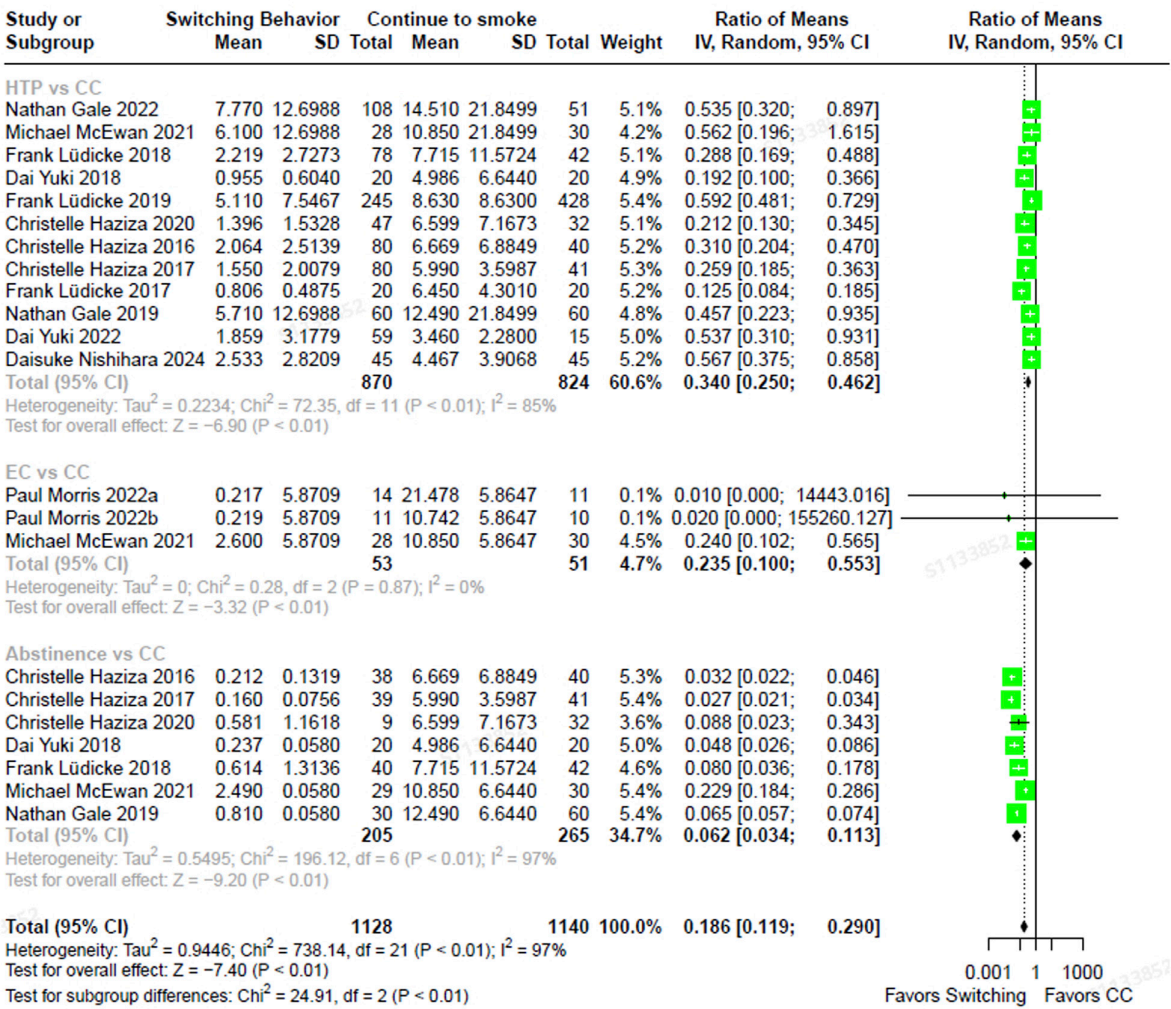
The forest plot illustrating the ratio of means in NNN between heated tobacco product switchers, electronic cigarette switchers, smoking abstainers, versus continued combustible cigarette smokers. NNN = N-Nitrosonornicotine.

The total NNAL levels in individuals who had completely switched to ECs were 67.8% lower (RoM, 0.322; 95% CI, 0.130–0.798) than in those who were continuing to smoke CCs (Figure 2). In individuals who had completely switched to HTPs, NNAL levels were 47.3% lower (RoM, 0.527; 95% CI, 0.433–0.641) than in continuous CC smokers. Those who abstained from smoking exhibited NNAL levels that were 60.3% lower (RoM, 0.397; 95% CI, 0.339–0.466) compared to those who smoked CCs exclusively. The direction and magnitude of difference in NNAL did not vary significantly between each switching groups versus continued CC use (test for subgroup differences, χ^2^ = 5.24, p = 0.07). Similarly, NNN levels were 76.5% lower in individuals who had completely switched to ECs (RoM, 0.235; 95% CI, 0.100–0.553), 66.0% lower in those who had completely switched to HTPs (RoM, 0.340; 95% CI, 0.250–0.462), and 93.8% lower in those who abstained from smoking CCs (RoM, 0.062; 95% CI, 0.034–0.113), compared to smoking CCs continuously (Figure 3). In contrast, the magnitude of difference in NNN varied significantly among switching groups, with substantially lower levels observed in abstainers compared to continuous CC smokers (test for subgroup differences, $\chi^{2}$ = 24.91, *p* < 0.01).

#### 3.4.2 SVOCs

In comparison to the continuous use of CCs, significantly lower concentrations of MHBMA (Supplementary Figure S2), SPMA (Supplementary Figure S3), and HEMA (Supplementary Figure S4) were observed in individuals who had entirely transitioned to ECs (RoM_MHBMA_, 0.234; 95% CI_MHBMA_, 0.099–0.551; RoM_SPMA_, 0.031; 95% CI_SPMA_, 0.001–0.681; RoM_HEMA_, 0.461; 95% CI_HEMA_, 0.273–0.777), HTPs (RoM_MHBMA_, 0.166; 95% CI_MHBMA_, 0.111–0.248; RoM_SPMA_, 0.135; 95% CI_SPMA_, 0.082–0.223; RoM_HEMA_, 0.372; 95% CIHEMA, 0.336–0.410) or those who had stopped smoking without any assistance (RoM_MHBMA_, 0.093; 95% CI_MHBMA_, 0.075–0.115; RoM_SPMA_, 0.082; 95% CI_SPMA_, 0.058–0.116; RoM_HEMA_, 0.365; 95% CI_HEMA_, 0.323–0.413). Significantly lower levels were also recorded for CEMA (Supplementary Figure S5) and 3-HMPMA (Supplementary Figure S6) following a complete switch to HTPs (RoM_CEMA_, 0.155; 95% CI_CEMA_, 0.117–0.204; RoM_3-HMPMA_, 0.421; 95% CI_3-HMPMA_, 0.316–0.561) and smoking cessation (RoM_CEMA_, 0.121; 95% CI_CEMA_, 0.103–0.142; RoM_3-HMPMA_, 0.329; 95% CI_3-HMPMA_, 0.184–0.588). However, individuals who had transitioned entirely to ECs did not show lower levels in these biomarkers, with the RoM for CEMA being 0.286 (95% CI, 0.044–1.861) and for 3-HMPMA being 0.280 (95% CI, 0.045–1.721) compared to continuous CC smokers. Furthermore, both the full switching and abstinence groups demonstrated comparable lower levels in the biomarkers MHBMA, SPMA, HEMA, and CEMA when assessed in comparison with those of continuous CC smokers. One study reported substantially lower GAMA (Supplementary Figure S7) levels in full EC switchers compared to those who were continuing to smoke, with a RoM of 0.550 (95% CI, 0.450–0.670), yet no significant difference was found between full HTP switchers and continuous CC smokers (RoM, 1.148; 95% CI, 0.819–1.609). Additionally, neither full switching to ECs (RoM, 0.609; 95% CI, 0.256–1.449) nor full switching to HTPs (RoM, 0.876; 95% CI, 0.619–1.240) resulted in significantly lower levels of AAMA (Supplementary Figure S8) compared to those who continued smoking CCs. AAs.

Compared to those who continued smoking CCs, individuals who had completely switched to either ECs or HTPs, as well as those who abstained from smoking, exhibited statistically significantly lower levels of 1-AN (Supplementary Figure S9), 2-AN (Supplementary Figure S10), 4-ABP (Supplementary Figure S11), and o-Tol (Supplementary Figure S12). The RoMs along with their corresponding 95% CIs were as follows: for EC switchers, RoM_2-AN_ was 0.056 (95% CI, 0.018–0.168), RoM_4-ABP_ was 0.117 (95% CI, 0.052–0.263), and RoM_o-Tol_ was 0.203 (95% CI, 0.123–0.335); for HTP switchers, RoM_1-AN_ was 0.054 (95% CI, 0.034–0.087), RoM_2-AN_ was 0.155 (95% CI, 0.114–0.211), RoM_4-ABP_ was 0.216 (95% CI, 0.167–0.278), and RoM_o-Tol_ was 0.457 (95% CI: 0.373–0.559); and for abstainers, RoM_1-AN_ was 0.053 (95% CI, 0.032–0.086), RoM_2-AN_ was 0.147 (95% CI, 0.109–0.200), RoM_4-ABP_ was 0.166 (95% CI, 0.134–0.206), and RoM_o-Tol_ was 0.431 (95% CI, 0.346–0.537). The direction and magnitude of effect sizes for 1-AN, 2-AN, and 4-ABP exposure were consistent across all switching groups compared to continuous CC smoking. Nonetheless, complete switching to ECs resulted in a more significant improvement in o-Tol concentrations versus continuous CC smoking than the other switching groups.

#### 3.4.3 PAHs

The full transition to HTPs (RoM, 0.406; 95% CI, 0.316–0.521) and the achievement of smoking abstinence (RoM, 0.296; 95% CI, 0.248–0.353) were associated with statistically significantly lower levels of 3-OH-B [a]P exposure when compared to continuous CC smoking (Supplementary Figure S13). While both switching groups exhibited favorable trends, the improvement in 3-OH-B [a]P levels was statistically more pronounced in the abstinence group than in the HTP switching group in relation to ongoing CC use (test for subgroup differences, $\chi^{2}$ = 4.11, *p* = 0.04). Supplementary Figure S14 depicted the pooled RoMs with 95% CIs for all 14 biomarkers, stratified by intervention group comparisons.

### 3.5 Baseline imbalance

There were no baseline imbalances in any intervention group comparisons for 1-AN, 2-AN, 4-ABP, AAMA, CEMA, GAMA, NNAL, o-Tol, or SPMA (Supplementary Table S2). However, compared to continuous CC smokers, HTP switchers exhibited significantly higher baseline levels of 3-HMPMA (RoM, 1.086; 95% CI, 1.014–1.164), 3-OH-B [a]P (RoM, 1.173; 95% CI, 1.051–1.309), MHBMA (RoM, 1.230; 95% CI, 1.085–1.393), and NNN (RoM, 1.170; 95% CI, 1.017–1.346). Similarly, abstainers showed statistically significantly higher baseline HEMA levels (RoM, 1.136; 95% CI, 1.063–1.240) than continuing CC smokers. Substantial heterogeneity was observed in baseline comparisons between EC switchers and continuous CC smokers for 3-HMPMA ( $I^{2}$ = 75.0%), AAMA ( $I^{2}$ = 79.0%), CEMA (I2 = 53.0%), and NNAL ( $I^{2}$ = 91.0%); between HTP switchers and continuous CC smokers for MHBMA ( $I^{2}$ = 91.0%); and between abstainers and continuous CC smokers for 3-OH-B [a]P ( $I^{2}$ = 92.0%). Some evidence of inconsistency was also observed for baseline AAMA ( $I^{2}$ = 21.0%) levels in the HTP switcher vs continuous CC smoker comparison and for baseline CEMA ( $I^{2}$ = 25.0%) levels in the abstainer vs CC smoker comparison.

### 3.6 Publication bias and sensitivity analysis

**FIGURE 4:**
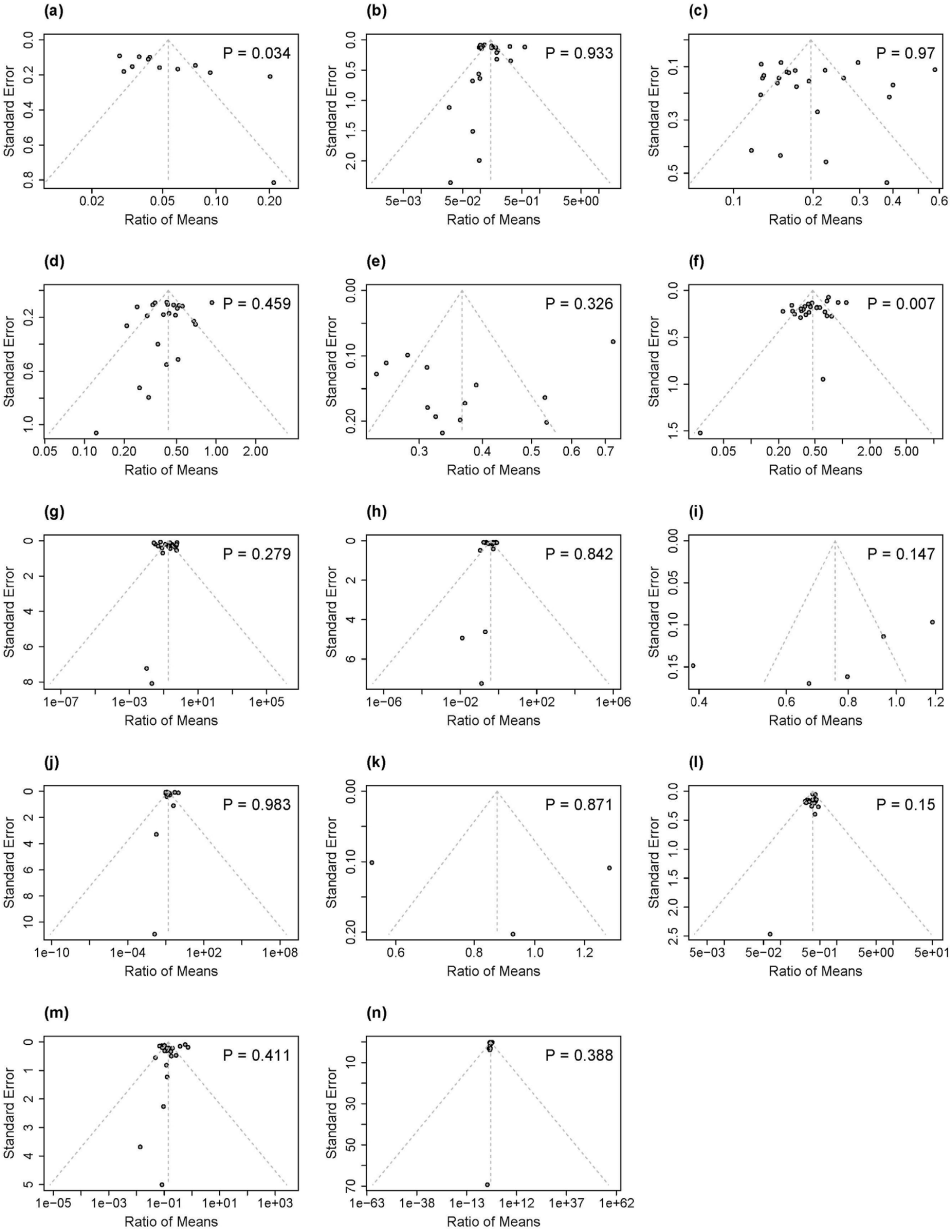
Funnel plots and P values for Egger's test for publication bias of included studies in our analysis associated with 14 biomarkers of exposure: **(a)** 1-AN, **(b)** 2-AN, **(c)** 4-ABP, **(d)** o-Tol, **(e)** 3-OH-B[a]P, **(f)** NNAL, **(g)** NNN, **(h)** 3-HMPMA, **(i)** AAMA, **(j)** CEMA, **(k)** GAMA, **(l)** HEMA, **(m)** MHBMA, **(n)** SPMA. 1-AN-1-aminonaphthalene; 2-AN = 2-aminonaphthalene; 4-ABP = 4-aminobiphenyl; o-Tolo-Toluidine; 3-OH-B[a]P = 3-Hydroxybenzo(a)pyrene; NNAL = total 4-(methylnitrosamino)-1-(3-pyridyl)-1-butanol; NNN = N-nitrosonornicotine; 3-HMPMA-3-hydroxy-1-methylpropylmercapturic acid; AAMA = acrylamide-mercapturic acid; CEMA 2-cyanoethylmercapturic acid; GAMA = glycidamide-mercapturic acid; HEMA = 2-hydroxyethyl-mercapturic acid; MHBMA = monohydroxybutenyl mercapturic acid; SPMA S-phenyl-mercapturic acid.

Each outcome variable was illustrated in funnel plots and subsequently assessed using Egger’s tests, as shown in Figure 4. The p-values from Egger’s test for 2-AN, 4-ABP, o-Tol, 3-OH-B [a]P, NNN, 3-HMPMA, AAMA, CEMA, GAMA, HEMA, MHBMA, and SPMA ranged from 0.147 to 0.983, indicating no evidence of publication bias. In contrast, the funnel plots for 1-AN and NNAL displayed notable asymmetry, confirmed by significant Egger’s test p-values of 0.034 for 1-AN and 0.007 for NNAL, suggesting the existence of publication bias for these specific biomarkers. Sensitivity analysis excluding individual studies with imputed SDs showed consistent results across all biomarkers (Supplementary Figure S15), except for GAMA in the HTP versus CC comparison and SPMA in the EC versus CC comparison. In these cases, only one study remained, precluding meta-analysis. The single study for GAMA indicated significantly higher exposure levels in full HTP switcher compared to continuing CC smokers while the single study for SPMA demonstrated comparable exposure levels between full EC switchers and continuing CC smokers.

## 4 Discussion

The global tobacco landscape has witnessed a dramatic transformation with the introduction of potential harm-reduction products, particularly ECs and HTPs. Manufacturers claim that these devices provide an alternative method of nicotine delivery while significantly reducing harm and risk compared to traditional CCs. Health risk estimation can be achieved by linking individual BoEs to specific health consequences, such as cancers, arising from exposure to harmful toxicants in tobacco smoke or aerosol. Our meta-analyses, which combined 18 randomized controlled trials involving smokers using novel tobacco products, indicated that a complete switch to either ECs or HTPs was associated with significantly lower levels of numerous carcinogenic biomarkers compared to ongoing CC use. This finding aligns with a growing body of literature and the scientific consensus that ECs and HTPs may function as reduced-risk alternatives (Eaton et al., 2018; McNeill et al., 2022; Kusonic et al., 2023; Liu et al., 2024), exposing users to a lower concentration of carcinogens than those who smoke CCs exclusively.

In this review, we identified a total of 12 BoEs that were available for comparative analysis between the abstinence group and the continuing CC group. Individuals who abstained from smoking demonstrated substantially reduced levels across all 12 BoEs compared to CC participants. Moreover, we observed minimal statistical heterogeneity, almost 0%, among the included studies for each BoE, underscoring the robustness and consistency of the evidence. Notably, among participants who completely switched to HTPs, the degree of reductions in 11 out of 12 BoEs were statistically comparable to those observed in the abstinence group. Similarly, for those who switched entirely to ECs, 7 out of 10 BoEs showed comparable reductions. These findings suggest that, in terms of exposure to specific toxicants, the use of HTPs and ECs may result in reductions similar to those achieved through complete smoking cessation. However, this does not imply that the overall health risks of ECs or HTPs are equivalent to those of smoking cessation, nor does it mean that these novel tobacco products are completely safe.

Our research revealed inconsistencies and, in some cases, adverse effects on BoEs for carcinogens among individuals who had completely switched from CC use to HTPs or ECs. Specifically, individuals who had fully adopted HTPs were not exposed to lower concentrations of the biomarkers GAMA and AAMA compared to those who were continuing with exclusive CC smoking. Likewise, smokers who switched to using ECs exclusively showed no significant differences in levels of CEMA, 3-HMPMA, or AAMA compared to exclusive CC smokers. In contrast, data synthesized from two RCTs indicated that complete EC switchers had significantly reduced levels of o-Tol compared to both complete HTP switchers and smoking abstainers, with CC-only users serving as the reference group. However, the scarcity of data on BoE outcomes in EC switchers has made it impossible to draw definitive conclusions. Furthermore, the presence of residual biomarkers in the human body remains a critical concern, regardless of whether biomarker levels are elevated or diminished in the intervention groups compared to exclusive CC smoking. Additional studies are necessary in order to better understand the health impacts of HTPs and ECs on the aforementioned BoEs, as well as investigate the implications of residual biomarker levels on individual health outcomes.

Our review has several limitations. First, 16 out of 17 included studies were funded by tobacco companies. The prevalence of industry-sponsored research is likely driven by the industry’s need to conduct costly clinical trials on biomarkers to meet regulatory requirements, such as the Premarket Tobacco Product Application (PMTA) in the United States (U.S. Food and Drug Administration, 2023). Such a funding landscape introduces a systematic risk of reporting bias (Soule et al., 2025) that may not be fully mitigated by standard ethical approval from Institutional Review Boards (IRBs) or rigorous peer review. For instance, sponsors may utilize study designs that favor their products, such as using only continuing smokers as a comparator, limiting follow-up periods to capture only the initial rapid decline in biomarkers, or selectively reporting statistically favorable outcomes. The single non-industry-funded study in our review (Hatsukami et al., 2020) did report finding consistent with our pooled analysis: smokers who switched completely to EC achieved greater reductions in toxicant exposure than continuing smokers, with most biomarker reduced levels comparable to those of abstainers within 8 weeks. However, this one study is insufficient to counteract concerns of widespread industry bias. Further research from independent institutions is critically needed to substantiate the harm reduction potential of switching from CC to newer-generation tobacco products.

Second, and likely related to the funding issue, our analysis revealed evidence of publication bias for two key biomarkers: 1-AN (Egger’s test, *p* = 0.034) and NNAL (Egger’s test, *p* = 0.007). The observed funnel plot asymmetries suggest that studies showing smaller or non-significant reduction effects for these BoEs after switching away from CCs to ECs or HTPs may be underrepresented in the published literature. Consequently, the true magnitude of exposure reduction for these BoEs is likely more modest than our pooled estimates indicate, further emphasizing the need for independent studies as well.

Third, the follow-up durations of the included studies varied considerably. The majority of trials (10 of 17) were short-term, with follow-up periods of 5–7 days. The remaining seven studies were longer-term, with durations ranging from 14 days to 360 days. While adequate for measuring BoEs with short half-lives (e.g., <25 h for SVOCs) (Yach and Scherer, 2023; Albiach-Delgado et al., 2022; Ashley et al., 2024), most short-term confinement studies are likely insufficient to capture the full trajectory of change for BoEs with longer half-lives, such as NNAL (10–45 days) (Yach and Scherer, 2023). Highly controlled nature of these studies may also suppress smokers’ urges by removing them from their usual environments, thus not reflecting real-world usage patterns and inflating the observed benefits of switching. For example, these trails often fail to account for the complexities of dual use—a common behavior where alternative nicotine products are used alongside CCs—which can confound harm reduction assessments. Indeed, evidence indicates that only substantial reductions ( ≥ 50%) in CC consumptions during transitions to ECs correlated with improved biomarker outcomes (Anic et al., 2022; Holt et al., 2023). Future research must therefore incorporate longer follow-up periods and longitudinal designs to better track real-world behaviors.

Furthermore, the generalizability of our findings is constrained by significant heterogeneity across both study populations and the products investigated. Most trials focused on healthy adult smokers in high-income countries, limiting applicability to populations with pre-existing health conditions or those in low- and middle-income countries (LMICs), where smoking prevalence and use patterns may differ substantially. The variability in the products themselves—spanning different device types, flavors, and nicotine strengths—also makes it difficult to isolate the effects of specific product characteristics on biomarker exposure. Future research should prioritize the inclusion of more diverse populations and incorporate designs that can parse the distinct effects of device type, flavoring, and nicotine strength on biomarker exposure.

Finally, our analysis was restricted to study-level data, which limited our ability to fully address baseline imbalances and the impact of our SD imputation strategy. Although pronounced improvements in BoEs among HTP users compared to continuing CC participants were still observed when HTP users had higher baseline BoE levels, these findings should be interpreted cautiously due to this limitation. On top of that, imputing the maximum SD from comparable studies could either down-weights or up-weights the study’s contribution and shift the pooled estimate to the null or to be more significant. While our sensitivity analysis excluding these studies did not alter the conclusions drastically, this remains a limitation. Access to individual participant data (IPD) would be essential for further exploring the relationships between EC or HTP use patterns, biomarkers of exposure, and health effects across a diverse range of populations.

Our review offers insight into the potential for harm reduction when completely switching from traditional CC smoking to novel tobacco product use, including ECs and HTPs, as indicated by biomarker levels. Considering the limitations discussed, there remains a need for further research involving larger cohorts over extended periods to better account for complex usage patterns and smoking behaviors. This will help to fully establish the potential health benefits of these products among experienced CC smokers.

## 5 Conclusion

Our systematic review and meta-analysis highlighted that individuals who completely switched from CCs to either ECs or HTPs exhibited a markedly lower exposure to a diverse array of carcinogenic HPHCs. Relative to continued smoking, the degree of reduction for most toxicants in these switchers approached that of complete abstainers. This finding, however, must be interpreted with caution. The persistence of certain toxicants at levels comparable to smoking CCs continuously, combined with the short-term follow-up periods (typically 5–7 days) of the included trials, suggest that while ECs and HTPs devices may represent a significant harm reduction step, they should not be considered risk-free. Future research is therefore essential to investigate the long-term health impacts of residual nicotine and tobacco-specific carcinogenic exposures following a switch from CC smoking to EC or HTP use.

## Abbreviations

HPHC, Harmful and potentially harmful constituent; EC, Electronic cigarette; HTP, Heated tobacco product; BoE, Biomarker of exposure; CC, Combustible cigarette; RCT, Randomized controlled trial; RoM, Ratio of means; COPD, Chronic obstructive pulmonary disease; WHO, World Health Organization; NCCDPHP, National Center for Chronic Disease Prevention and Health Promotion; ANDS, Alternative nicotine delivery system; EUR, European Region; WPR, Western Pacific Region; AMR, Americas Region; AFR, African Region; NRT, Nicotine replacement therapy; PRISMA, Preferred Reporting Items for Systematic Reviews and Meta-Analyses; ROB 2, Version 2 of the Cochrane risk-of-bias tool for randomized trials; ANCOVA, Analysis of covariance; LMEM, Linear mixed-effect model; CI, Confidence interval; TSNA, Tobacco-specific nitrosamine; SVOC, Semi-volatile organic compound; AA, Aromatic amine; PAH, Polycyclic aromatic hydrocarbon; IARC, International Agency for Research on Cancer; PMTA, Premarket Tobacco Product Application.
