# Supplementary material for: Assessing biomarkers of exposure to carcinogens associated with combustible cigarettes, electronic cigarettes, and heated tobacco products: a systematic review and meta-analysis
Source: Front Pharmacol. 2025 Jul 29;16:1630961. doi: 10.3389/fphar.2025.1630961 (PMC12339566; doi:10.3389/fphar.2025.1630961)
Supplement: Supplementary file 1 [file DataSheet1.docx]

Supplementary Material

# Supplementary Tables

**Supplementary Table 1**. Summary of Biomarkers and Associated HPHCs Assessed in Study.

| **Chemical Category** | **Biomarker** | **Biomarker Matrix** | **Chemical Name** | **Associated HPHC** | **Risk Association^a^** |
| --- | --- | --- | --- | --- | --- |
| Tobacco-specific  Nitrosamines | Total NNAL | Urine | 4-(methylnitrosamino)-1-(3-pyridyl)-1-butanol | NNK^1,2,3^ | CA |
|  | NNN | Urine | N-nitrosonornicotine | NNN^1,2,3^ | CA |
| Semi-volatile  Organics  (Mercapturic Acids) | CEMA | Urine | 2-cyanoethylmercappturic acid | Acrylonitrile^1,2^ | CA, RT |
|  | 3-HMPMA | Urine | 3-hydroxy-1-methylpropyl-mercapturic acid | Crotonaldehyde^1,2^ | CA |
|  | MHBMA | Urine | Monohydroxylbutenyl-mercapturic acid | 1,3-butadiene^1,2,3^ | CA, RT, RDT |
|  | SPMA | Urine | N-Acetyl-S-phenyl-l-cysteine, S-phenyl-mercapturic acid | Benzene^1,2,3^ | CA, RT, RDT |
|  | AAMA | Urine | N-Acetyl-S-(2-carbamoylethyl)-l-cysteine, acrylamide-mercapturic acid | Acrylamide^1^ | CA |
|  | GAMA | Urine | N-Acetyl-S-(2-carbamoylethyl-2-hydroxy)-l-cysteine, glycidamide-mercapturic acid | Acrylamide^1^ | CA |
|  | HEMA | Urine | 2-hydroxyethyl-mercapturic acid | Ethylene oxide^1^ | CA, RT, RDT |
| Aromatic Amines | 1-AN | Urine | 1-aminonaphthalene | 1-aminonaphthalene^1,2^ | CA |
|  | 2-AN | Urine | 2-aminonaphthalene | 2-aminonaphthalene^1,2^ | CA |
|  | 4-ABP | Urine | 4-aminobiphenyl | 4-aminobiphenyl^1,2^ | CA |
|  | o-Tol | Urine | Ortho-toluidine | o-toluidine^1,2^ | CA |
| Polycyclic aromatic  hydrocarbons | 3-OH-B[a]P | Urine | 3-Hydroxybenzo(a)pyrene | Benzo[a]pyrene (B[a]P) ^1,2,3^ | CA |

***Note.* Abbreviations.** HPHC, harmful and potentially harmful constituent; NNK, nicotine-derived nitrosamine ketone; CA, carcinogen; RDT, reproductive and developmental toxicant; AD, Addictive toxicant; RT, respiratory toxicant; CT, cardiovascular toxicant.

^1^Constituent included in FDA list of HPHCs in Tobacco Products and Tobacco Smoke (Federal Register, 2012).

^2^Constituent included in the PMTA ENDS Draft/Final Guidance (FDA, 2016/2019).

^3^Constituent included in WHO list of toxicants recommended for mandated lowering.

^a^Adapted from FDA Draft Guidance, “Harmful and Potentially Constituents in Tobacco Products and Tobacco Smoke: Established List”, Accessed on FDA.gov, October 1, 2020.

**Supplementary Table 2**. Results for comparisons on biomarkers of exposure to carcinogens between different switching behaviors and continued combustible cigarette (CC) smoking at baseline from studies with available baseline data.^*^

| Biomarker | Comparison | No. studies (participants) |  | Fixed-effect meta-analysis | |
| --- | --- | --- | --- | --- | --- |
|  |  | Data available |  | Ratio of Mean (95% CI) | Heterogeneity |
| 1-AN | HTP switchers vs. CC smokers | 6 (649) |  | 1.047 (0.961, 1.139) | Q = 4.85, *p* = 0.43, *I*^2^ = 0.0% |
|  | CC abstainers vs. CC smokers | 5 (321) |  | 0.969 (0.862, 1.088) | Q = 0.22, *p* = 0.99, *I*^2^ = 0.0% |
| 2-AN | HTP switchers vs. CC smokers | 12 (1126) |  | 1.041 (0.973, 1.114) | Q = 4.09, *p* = 0. 97, *I*^2^ = 0.0% |
|  | CC abstainers vs. CC smokers | 8 (526) |  | 0.984 (0.891, 1.088) | Q = 0.81, *p* = 1.00, *I*^2^ = 0.0% |
| 3-HMPMA | HTP switchers vs. CC smokers | 10 (1004) |  | 1.086 (1.014, 1.164) | Q = 3.17, *p* = 0.96, *I*^2^ = 0.0% |
|  | EC switchers vs. CC smokers | 2 (213) |  | 1.054 (0.929, 1.196) | Q = 3.94, *p* = 0.05, *I*^2^ = 75.0% |
|  | CC abstainers vs. CC smokers | 4 (281) |  | 0.965 (0.855, 1.089) | Q = 0.09, *p* = 0.99, *I*^2^ = 0.0% |
| 3-OH-B[a]P | HTP switchers vs. CC smokers | 6 (604) |  | 1.173 (1.051, 1.309) | Q = 2.65, *p* = 0.75, *I*^2^ = 0.0% |
|  | CC abstainers vs. CC smokers | 5 (321) |  | 0.633 (0.564, 0.710) | Q = 48.42, *p* < 0.01, *I*^2^ = 92.0% |
| 4-ABP | HTP switchers vs. CC smokers | 12 (1126) |  | 1.049 (0.978, 1.126) | Q = 4.75, *p* = 0.94, *I*^2^ = 0.0% |
|  | CC abstainers vs. CC smokers | 8 (526) |  | 0.979 (0.879, 1.091) | Q = 2, *p* = 0.96, *I*^2^ = 0.0% |
| AAMA | HTP switchers vs. CC smokers | 3 (241) |  | 1.092 (0.934, 1.277) | Q = 2.53, *p* = 0.28, *I*^2^ = 21.0% |
|  | EC switchers vs. CC smokers | 2 (212) |  | 0.981 (0.875, 1.099) | Q = 4.8, *p* = 0.03, *I*^2^ = 79.0% |
| CEMA | HTP switchers vs. CC smokers | 10 (981) |  | 1.035 (0.963, 1.113) | Q = 3.75, *p* = 0.93, *I*^2^ = 0.0% |
|  | EC switchers vs. CC smokers | 2 (213) |  | 1.049 (0.932, 1.180) | Q = 2.12, *p* = 0.15, *I*^2^ = 53.0% |
|  | CC abstainers vs. CC smokers | 7 (470) |  | 0.991 (0.918, 1.070) | Q = 7.99, *p* = 0.24, *I*^2^ = 25.0% |
| GAMA | HTP switchers vs. CC smokers | 2 (183) |  | 1.091 (0.937, 1.269) | Q = 0.01, *p* = 0.91, *I*^2^ = 0.0% |
| HEMA | HTP switchers vs. CC smokers | 9 (941) |  | 1.018 (0.902, 1.150) | Q = 3.33, *p* = 0.91, *I*^2^ = 0.0% |
|  | CC abstainers vs. CC smokers | 7 (470) |  | 1.136 (1.063, 1.240) | Q = 32.93, *p* < 0.01, *I*^2^ = 82% |
| MHBMA | HTP switchers vs. CC smokers | 12 (1126) |  | 1.230 (1.085, 1.393) | Q = 117.28, *p* < 0.01, *I*^2^ = 91.0% |
|  | CC abstainers vs. CC smokers | 8 (526) |  | 0.899 (0.737, 1.097) | Q = 4.08, *p* = 0.77, *I*^2^ = 0.0% |
| NNAL | HTP switchers vs. CC smokers | 12 (1125) |  | 1.048 (0.951, 1.154) | Q = 5.53, *p* = 0.90, *I*^2^ = 0.0% |
|  | EC switchers vs. CC smokers | 2 (211) |  | 0.888 (0.773, 1.020) | Q = 11.23, *p* < 0.01, *I*^2^ = 91.0% |
|  | CC abstainers vs. CC smokers | 7 (444) |  | 0.977 (0.854, 1.118) | Q = 1.64, *p* = 0.95, *I*^2^ = 0.0% |
| NNN | HTP switchers vs. CC smokers | 10 (981) |  | 1.170 (1.017, 1.346) | Q = 5.25, *p* = 0.81, *I*^2^ = 0.0% |
|  | CC abstainers vs. CC smokers | 7 (470) |  | 1.082 (0.880, 1.331) | Q = 2.12, *p* = 0.91, *I*^2^ = 0.0% |
| o-Tol | HTP switchers vs. CC smokers | 12 (1121) |  | 1.031 (0.961, 1.105) | Q = 8.73, *p* = 0.65, *I*^2^ = 0.0% |
|  | CC abstainers vs. CC smokers | 8 (523) |  | 0.964 (0.871, 1.066) | Q = 5.03, *p* = 0.66, *I*^2^ = 0.0% |
| SPMA | HTP switchers vs. CC smokers | 12 (1126) |  | 1.082 (0.969, 1.208) | Q = 8.85, *p* = 0.64, *I*^2^ = 0.0% |
|  | CC abstainers vs. CC smokers | 8 (526) |  | 0.948 (0.810, 1.108) | Q = 4.25, *p* = 0.75, *I*^2^ = 0.0% |

*Note*. 1-AN = 1-aminonaphtalene; 2-AN = 2-aminonaphtalene; 3-HMPMA = 3-hydroxy-1-methylpropylmercapturic acid; 3-OH-B[a]P = 3-Hydroxybenzo(a)pyrene; 4-ABP = 4-aminobiphenyl; AAMA = acrylamide-mercapturic acid; CEMA = 2-cyanoethylmercapturic acid; GAMA = glycidamide-mercapturic acid; HEMA = 2-hydroxyethyl-mercapturic acid; MHBMA = monohydroxybutenyl mercapturic acid; NNAL = total 4-(methylnitrosamino)-1-(3-pyridyl)-1-butanol; NNN = N-nitrosonornicotine; o-Tol = o-Toluidine; SPMA = S-phenyl-mercapturic acid.

^*^ Fixed-effect meta-analysis comparing baseline BoE levels of EC switchers or CC abstainers to those of continuing CC smokers was not performed because no study or only one study was available for these analyses for each BoE.

# Supplementary Figures

**
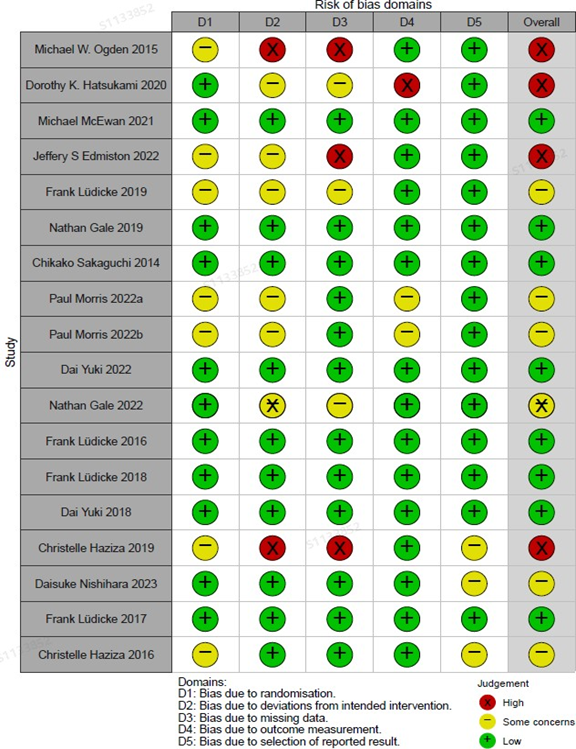
**

**(a)**

**
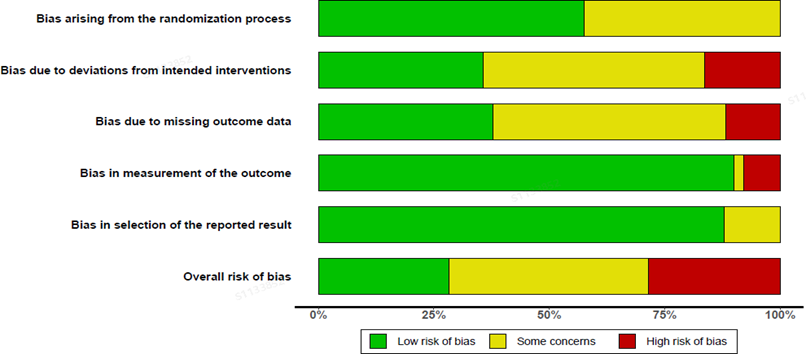
**

**(b)**

**Supplementary Figure 1.** Risk of bias assessment is presented in (a) a traffic light plot and (b) a summary plot according to the Cochrane risk-of-bias 2.0 tool, created using robvis package in R Software.

**
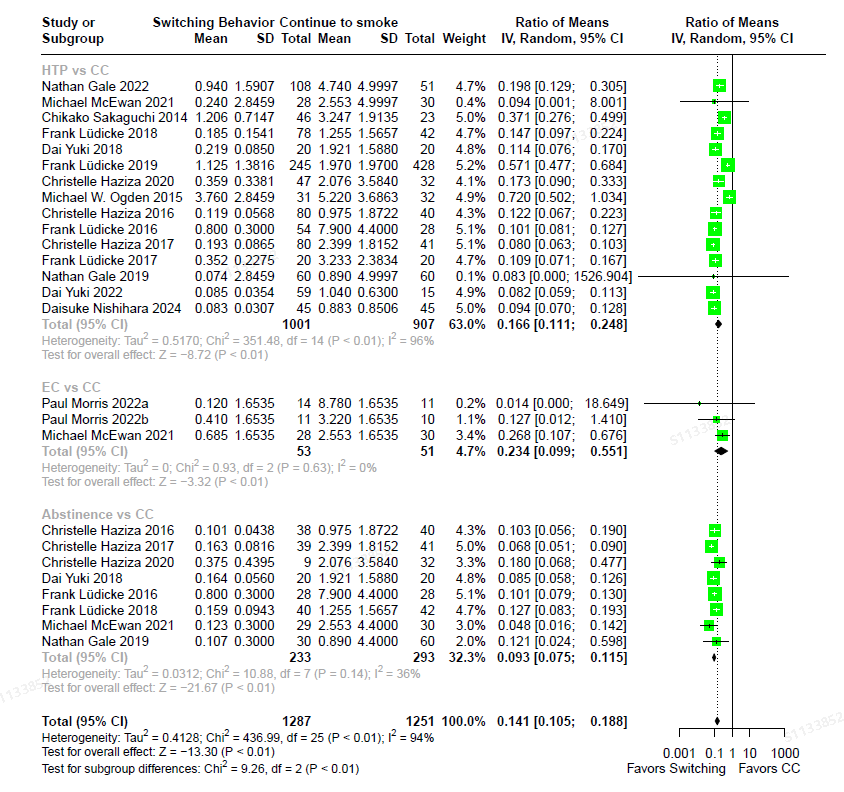
**

**Supplementary Figure 2.** The forest plot illustrating the ratio of means in MHBMA between heated tobacco product switchers, electronic cigarette switchers, smoking abstainers, versus continued combustible cigarette smokers. MHBMA = monohydroxybutenyl mercapturic acid.

**
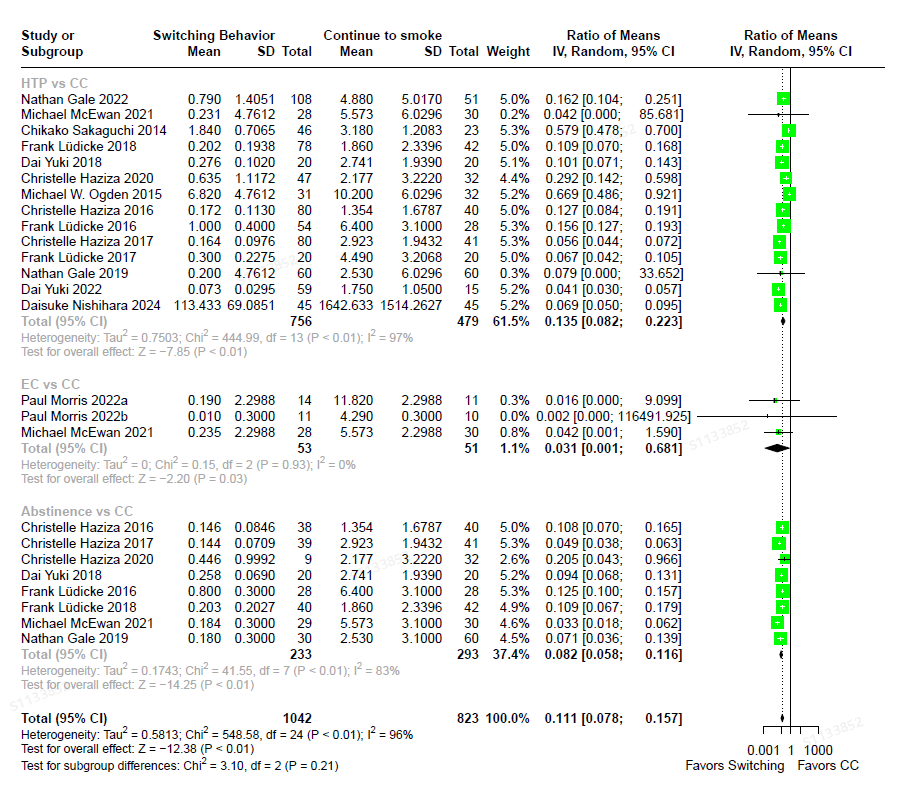
**

**Supplementary Figure 3.** The forest plot illustrating the ratio of means in SPMA between heated tobacco product switchers, electronic cigarette switchers, smoking abstainers, versus continued combustible cigarette smokers. SPMA = S-phenyl-mercapturic acid.


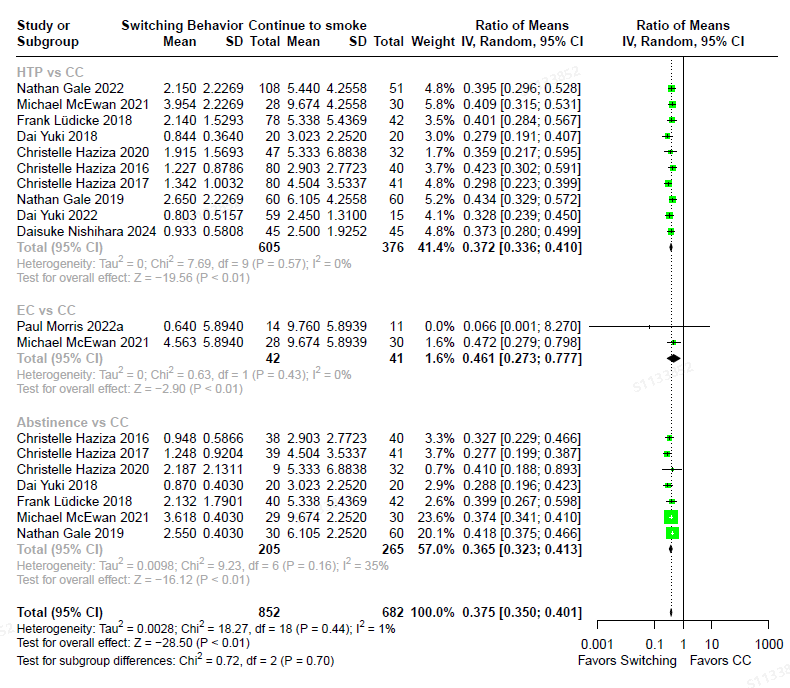


**Supplementary Figure 4.** The forest plot illustrating the ratio of means in HEMA between heated tobacco product switchers, electronic cigarette switchers, smoking abstainers, versus continued combustible cigarette smokers. HEMA = 2-hydroxyethyl-mercapturic acid.


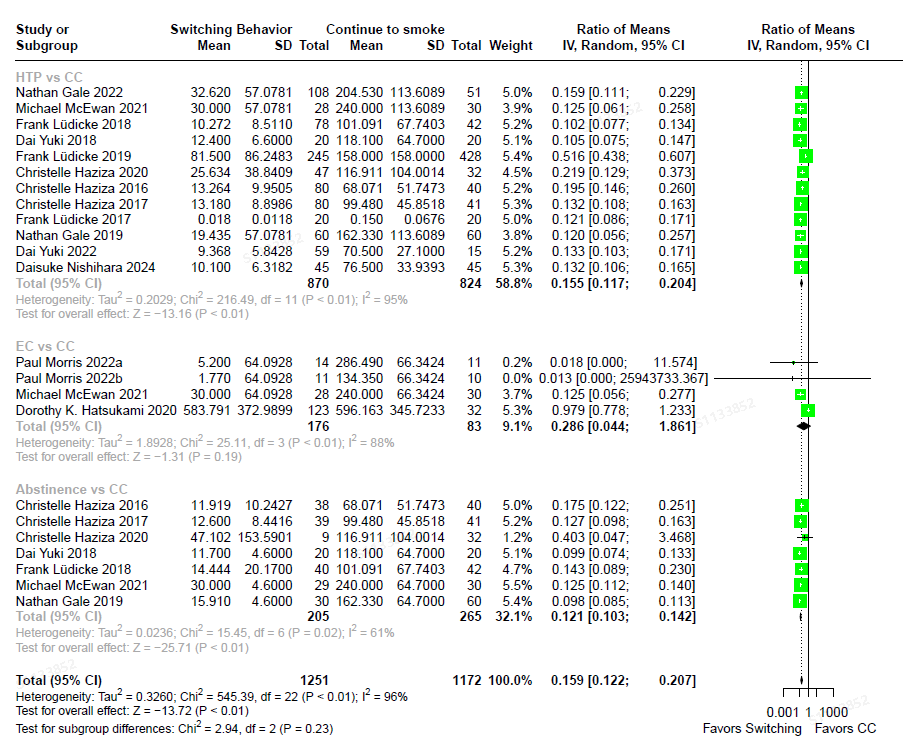


**Supplementary Figure 5.** The forest plot illustrating the ratio of means in CEMA between heated tobacco product switchers, electronic cigarette switchers, smoking abstainers, versus continued combustible cigarette smokers. CEMA = 2-cyanoethylmercapturic acid.


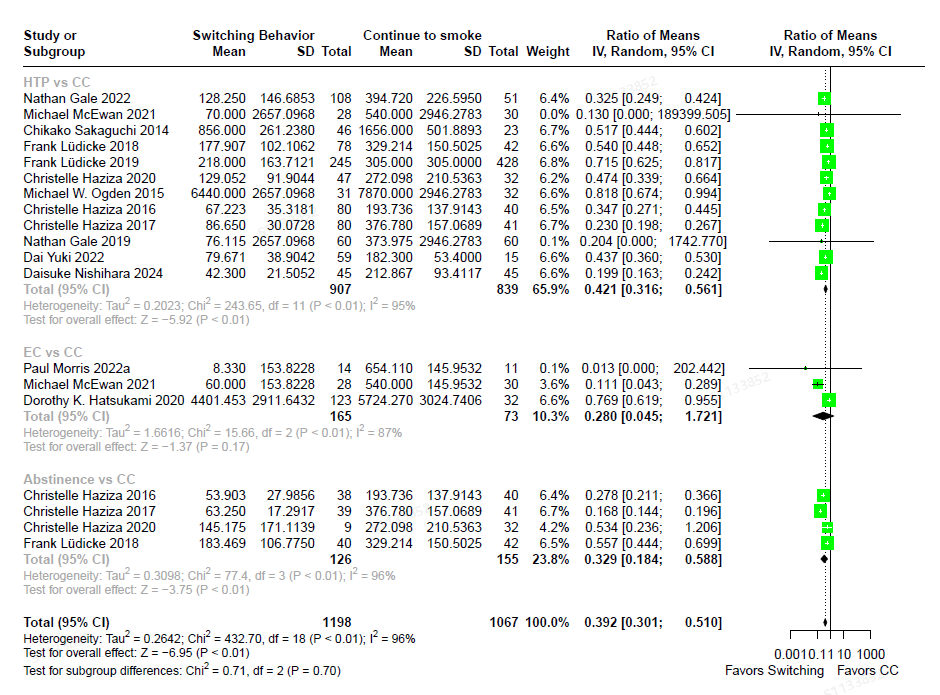


**Supplementary Figure 6.** The forest plot illustrating the ratio of means in 3-HMPMA between heated tobacco product switchers, electronic cigarette switchers, smoking abstainers, versus continued combustible cigarette smokers. 3-HMPMA = 3-hydroxy-1-methylpropylmercapturic acid.


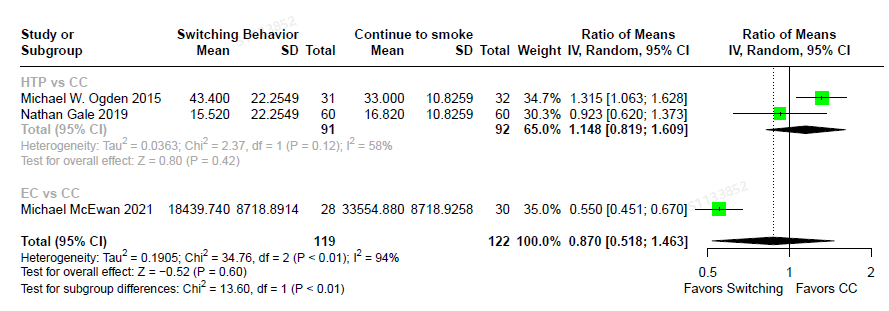


**Supplementary Figure 7.** The forest plot illustrating the ratio of means in GAMA between heated tobacco product switchers, electronic cigarette switchers, smoking abstainers, versus continued combustible cigarette smokers. GAMA = glycidamide-mercapturic acid.


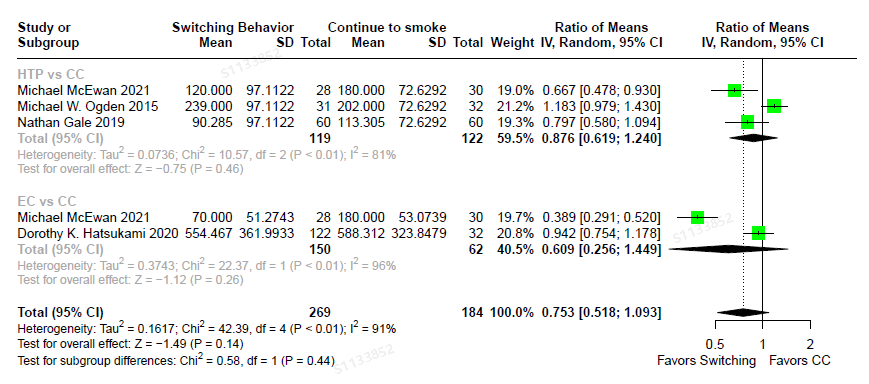


**Supplementary Figure 8.** The forest plot illustrating the ratio of means in AAMA between heated tobacco product switchers, electronic cigarette switchers, smoking abstainers, versus continued combustible cigarette smokers. AAMA = acrylamide-mercapturic acid.


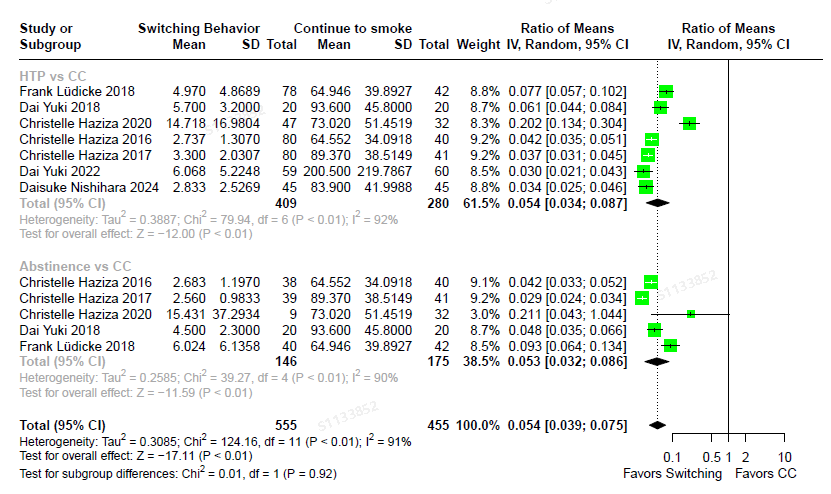


**Supplementary Figure 9.** The forest plot illustrating the ratio of means in 1-AN between heated tobacco product switchers, electronic cigarette switchers, smoking abstainers, versus continued combustible cigarette smokers. 1-AN = 1-aminonaphtalene.


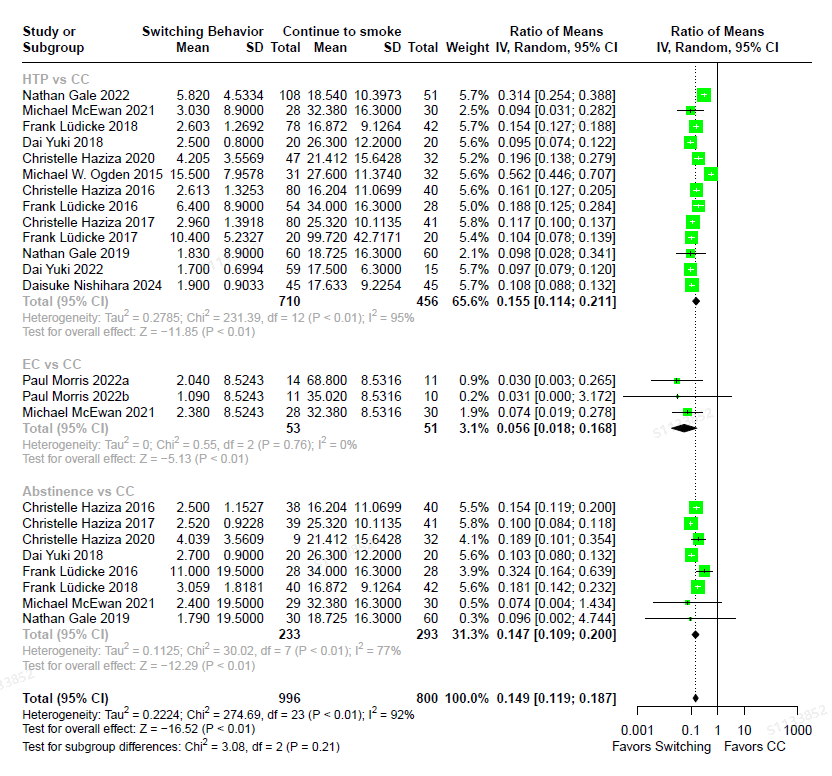


**Supplementary Figure 10.** The forest plot illustrating the ratio of means in 2-AN between heated tobacco product switchers, electronic cigarette switchers, smoking abstainers, versus continued combustible cigarette smokers. 2-AN = 2-aminonaphtalene.


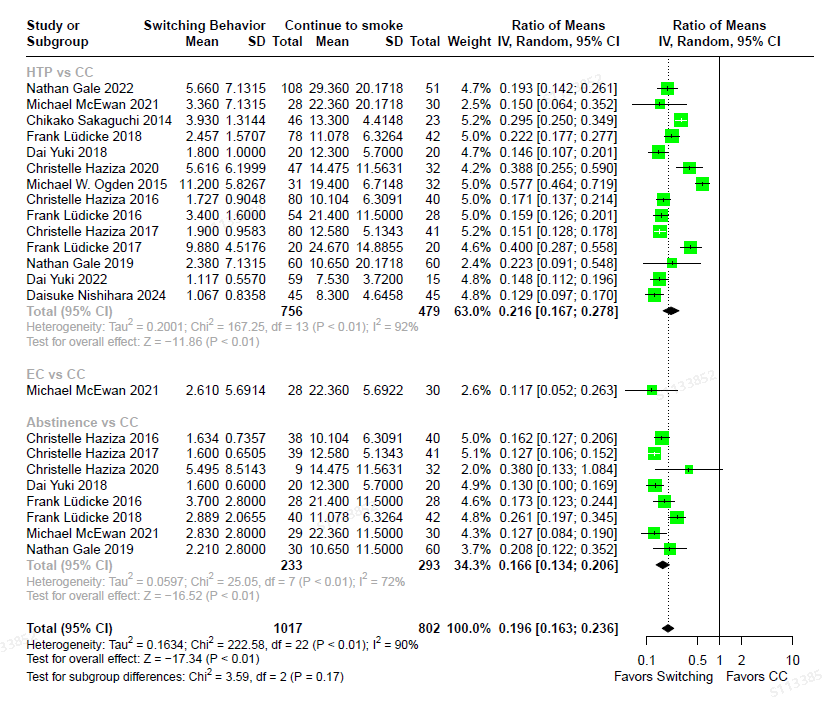


**Supplementary Figure 11.** The forest plot illustrating the ratio of means in 4-ABP between heated tobacco product switchers, electronic cigarette switchers, smoking abstainers, versus continued combustible cigarette smokers. 4-ABP = 4-aminobiphenyl.


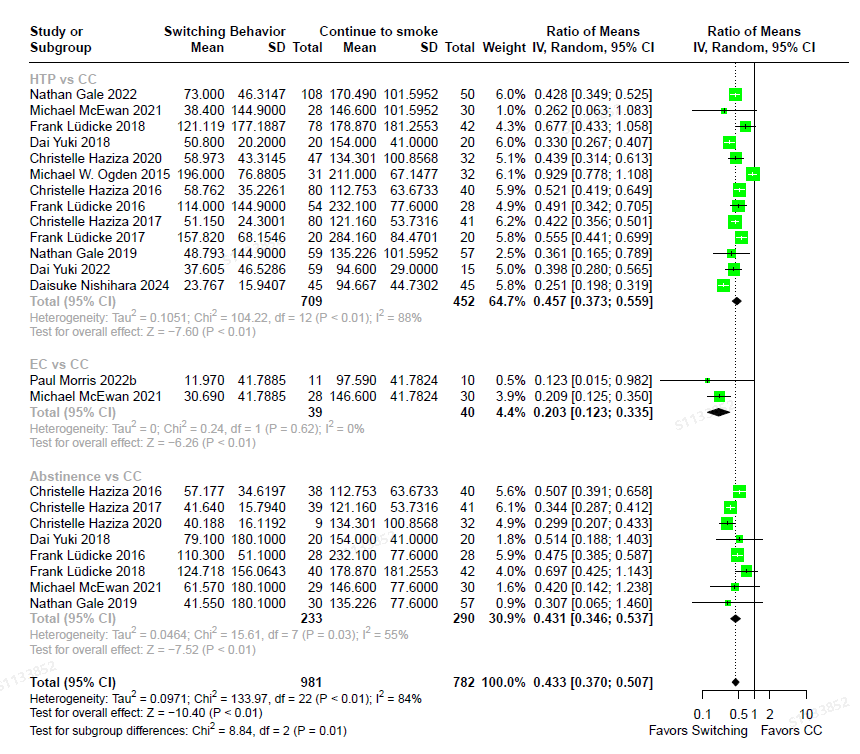


**Supplementary Figure 12.** The forest plot illustrating the ratio of means in o-Tol between heated tobacco product switchers, electronic cigarette switchers, smoking abstainers, versus continued combustible cigarette smokers. o-Tol = o-Toluidine.


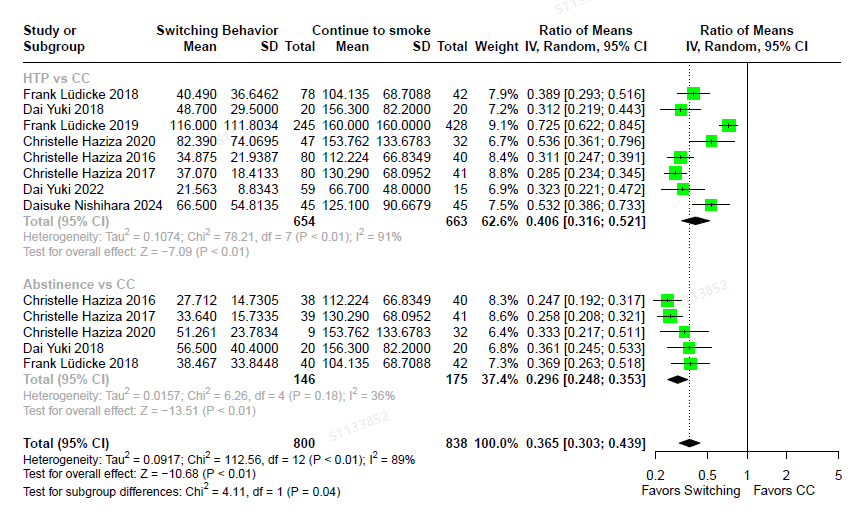


**Supplementary Figure 13.** The forest plot illustrating the ratio of means in 3-OH-B[a]P between heated tobacco product switchers, electronic cigarette switchers, smoking abstainers, versus continued combustible cigarette smokers. 3-OH-B[a]P = 3-Hydroxybenzo(a)pyrene.


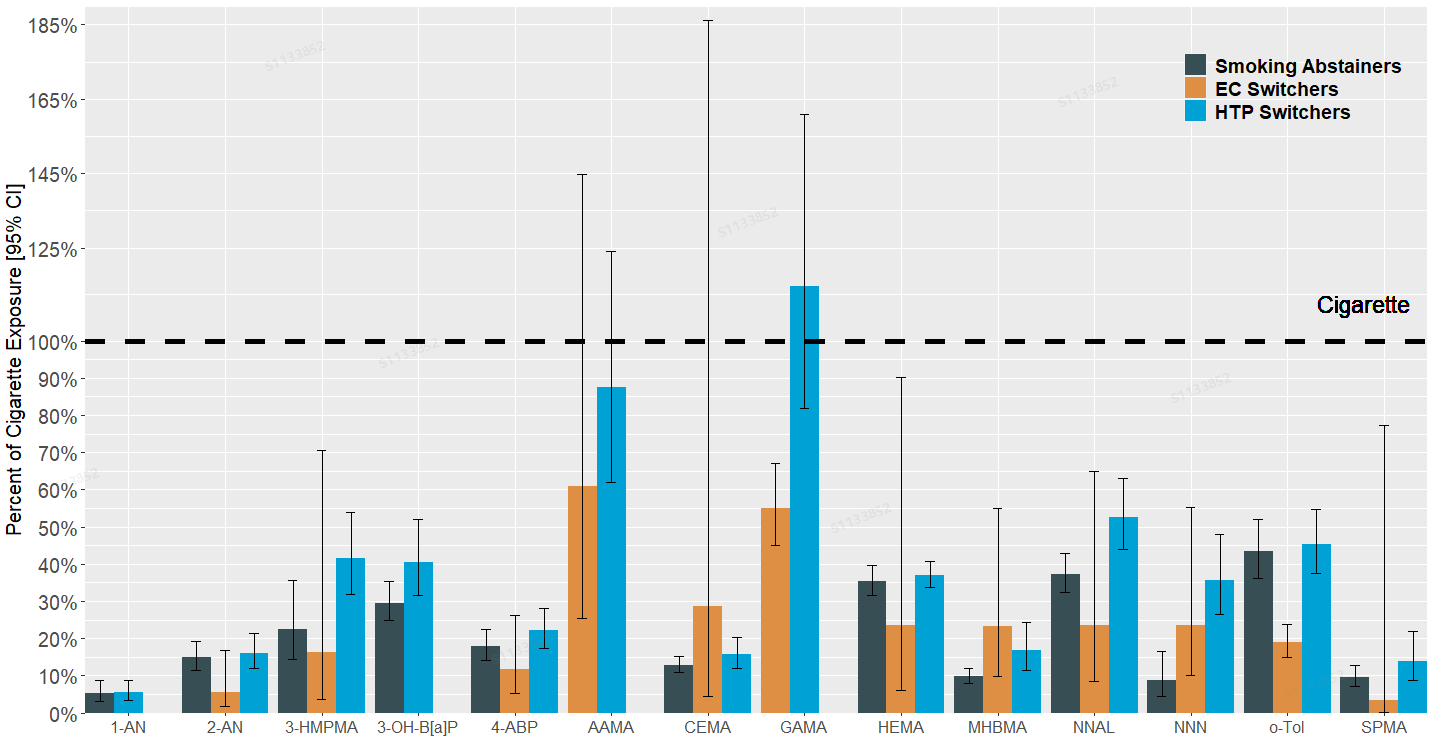


**Supplementary Figure 14.** Biomarkers of carcinogenic exposure among individuals who abstained from smoking, individuals who completely switched to EC use, individuals who completely switched to HTP use, compared to those who continued smoking CCs exclusively. 1-AN = 1-aminonaphtalene; 2-AN = 2-aminonaphtalene; 3-HMPMA = 3-hydroxy-1-methylpropylmercapturic acid; 3-OH-B[a]P = 3-Hydroxybenzo(a)pyrene; 4-ABP = 4-aminobiphenyl; AAMA = acrylamide-mercapturic acid; CEMA = 2-cyanoethylmercapturic acid; GAMA = glycidamide-mercapturic acid; HEMA = 2-hydroxyethyl-mercapturic acid; MHBMA = monohydroxybutenyl mercapturic acid; NNAL = total 4-(methylnitrosamino)-1-(3-pyridyl)-1-butanol; NNN = N-nitrosonornicotine; o-Tol = o-Toluidine; SPMA = S-phenyl-mercapturic acid.


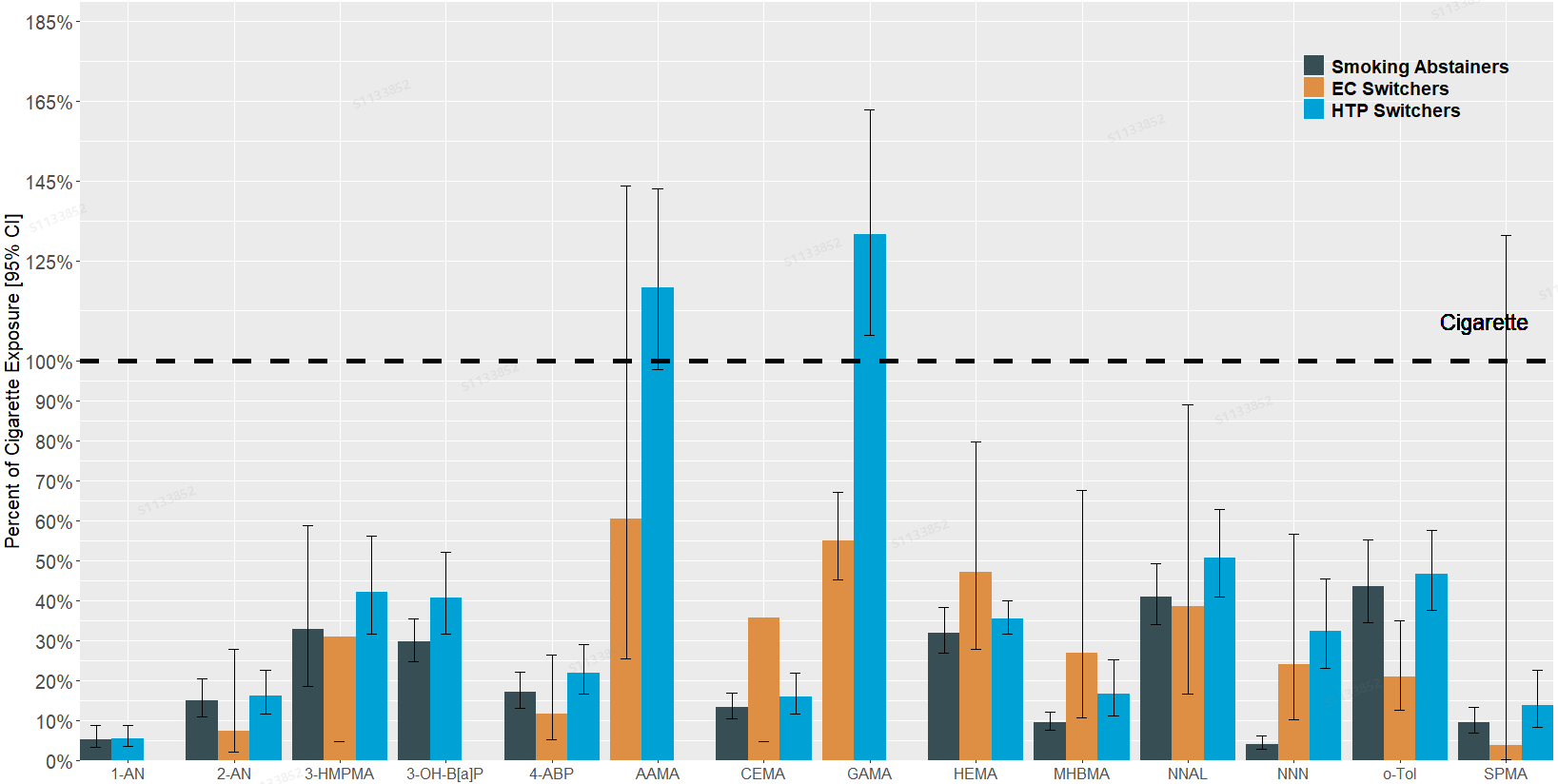


**Supplementary Figure 15.** Biomarkers of carcinogenic exposure among individuals who abstained from smoking, individuals who completely switched to EC use, individuals who completely switched to HTP use, compared to those who continued smoking CCs exclusively in sensitivity analysis. 1-AN = 1-aminonaphtalene; 2-AN = 2-aminonaphtalene; 3-HMPMA = 3-hydroxy-1-methylpropylmercapturic acid; 3-OH-B[a]P = 3-Hydroxybenzo(a)pyrene; 4-ABP = 4-aminobiphenyl; AAMA = acrylamide-mercapturic acid; CEMA = 2-cyanoethylmercapturic acid; GAMA = glycidamide-mercapturic acid; HEMA = 2-hydroxyethyl-mercapturic acid; MHBMA = monohydroxybutenyl mercapturic acid; NNAL = total 4-(methylnitrosamino)-1-(3-pyridyl)-1-butanol; NNN = N-nitrosonornicotine; o-Tol = o-Toluidine; SPMA = S-phenyl-mercapturic acid.
